# Deep learning model to predict Epstein–Barr virus associated gastric cancer in histology

**DOI:** 10.1038/s41598-022-22731-x

**Published:** 2022-11-02

**Authors:** Yeojin Jeong, Cristina Eunbee Cho, Ji-Eon Kim, Jonghyun Lee, Namkug Kim, Woon Yong Jung, Joohon Sung, Ju Han Kim, Yoo Jin Lee, Jiyoon Jung, Juyeon Pyo, Jisun Song, Jihwan Park, Kyoung Min Moon, Sangjeong Ahn

**Affiliations:** 1 Genome & Health Data Lab, School of Public Health, Seoul National University, Seoul, Republic of Korea; 2 Department of Convergence Medicine, Asan Medical Center, University of Ulsan College of Medicine, Seoul, Republic of Korea; 3 Wonkwang University Medical Research Convergence Center, Wonkwang University Hospital, Iksan, Republic of Korea; 4 Department of Medical and Digital Engineering, Hanyang University College of Engineering, Seoul, Republic of Korea; 5 Department of Pathology, Hanyang University Guri Hospital, Hanyang University College of Medicine, Guri, Republic of Korea; 6 Division of Biomedical Informatics, Seoul National University College of Medicine, Seoul National University Biomedical Informatics (SNUBI), Seoul, Republic of Korea; 7 Department of Pathology, Korea University Anam Hospital, Korea University College of Medicine, 73 Goryeodae‐ro, Seongbuk‐gu, Seoul, 02841 Republic of Korea; 8 Department of Pathology, Kangnam Sacred Heart Hospital, College of Medicine, Hallym University, Seoul, Republic of Korea; 9 Department of Pathology, International St. Mary’s Hospital, Catholic Kwandong University College of Medicine, Incheon, Republic of Korea; 10 Department of Pathology, Ewha Womans University Seoul Hospital, Ewha Womans University College of Medicine, Seoul, Republic of Korea; 11 School of Software Convergence, College of Software Convergence, Dankook University, Yongin-si, Republic of Korea; 12 Department of Pulmonary, Allergy, and Critical Care Medicine, Gangneung Asan Hospital, College of Medicine, University of Ulsan, 38, Bangdong-gil, Sacheon-myeon, Gangneung-si, 25440 Gangwon-do Republic of Korea

**Keywords:** Gastric cancer, Cancer screening, Predictive markers

## Abstract

The detection of Epstein–Barr virus (EBV) in gastric cancer patients is crucial for clinical decision making, as it is related with specific treatment responses and prognoses. Despite its importance, the limited medical resources preclude universal EBV testing. Herein, we propose a deep learning-based EBV prediction method from H&E-stained whole-slide images (WSI). Our model was developed using 319 H&E stained WSI (26 EBV positive; TCGA dataset) from the Cancer Genome Atlas, and 108 WSI (8 EBV positive; ISH dataset) from an independent institution. Our deep learning model, EBVNet consists of two sequential components: a tumor classifier and an EBV classifier. We visualized the learned representation by the classifiers using UMAP. We externally validated the model using 60 additional WSI (7 being EBV positive; HGH dataset). We compared the model’s performance with those of four pathologists. EBVNet achieved an AUPRC of 0.65, whereas the four pathologists yielded a mean AUPRC of 0.41. Moreover, EBVNet achieved an negative predictive value, sensitivity, specificity, precision, and F1-score of 0.98, 0.86, 0.92, 0.60, and 0.71, respectively. Our proposed model is expected to contribute to prescreen patients for confirmatory testing, potentially to save test-related cost and labor.

## Introduction

The Epstein–Barr virus (EBV) is present in approximately 10% of gastric cancer patients worldwide^1^, with the most prevalent among EBV-attributed malignancies^2,3^. The presence of EBV has been recognized as a potential biomarker for precision oncology in gastric cancers^4–8^. EBV-associated gastric cancers exhibit characteristic genetic and epigenetic alteration, which has multipronged effects on their unique phenotypes^9,10^. EBV-positive gastric cancer has high immunogenicity^11^ with overexpression of *PD-L1* and *PD-L2*^12,13^, clinically approved biomarkers for immune checkpoint inhibitors^14,15^. Deregulated immune response genes in this subtype of tumor affect the tumor immune microenvironment^16^, leading to a unique spatial arrangement of tumor cells within exuberant lymphoid stroma: so-called “lymphoepithelioma-like carcinoma”^17^. Prominent tumor-infiltrating lymphocytes, the characteristic morphologic feature in EBV-positive gastric cancer, can be a surrogate indicator of tumor behavior and prognosis^17–19^, associated with a lower frequency of lymph node metastasis^5–8,20,21^. With a low risk of lymph node metastasis in this subtype, early gastric cancers associated with EBV have been proposed as candidates for local excision regardless of the depth of the submucosal invasion^5–8,20,21^. Therefore, a revised criteria was proposed for endoscopic resection in patients with EBV-positive early gastric cancer^6,8,21^. Taken together, the identification of EBV status in gastric cancer, which links to a treatment-relevant phenotype, is important to provide effective therapeutic options and strategies^22^.

Manual microscopic inspection of H&E histology may be used to predict EBV status in gastric cancer, and may therefore have a role in screening EBV status for further confirmatory test. However, morphologic assessment has a disadvantage of low inter- and intra-rater agreement. A paramount diagnostic method for identifying a tumor as “EBV-positive” is the presence of EBV-encoded RNA in situ hybridization (EBER-ISH)^11,23^. However, the limitations on medical resources preclude universal testing of EBV status. Therefore, the development of a widely accessible and cost-effective tool for the EBV testing is essential.

As an unprecedented breakthrough in artificial intelligence technology, histology-based deep learning approaches are expected to identify novel biomarkers for oncology practice with a precision beyond human performance^24^. Prior studies have proven to facilitate learning of morphologic feature representation, correlating to molecular alterations, from digitized whole-slide images (WSI). They have inferred genetic traits including EBV status, actionable driving mutation, microsatellite instability, gene signature, and molecular tumor subtypes, in various malignant images (Supplementary Table S1 and S2)^25–51^. These studies shed light on the morphology-molecular association or “histo-genomics”^52,53^, which may contribute toward the discovery of cost-effective biomarkers and improved therapeutic options^54^.

In this study, we present a deep learning-based system for automated EBV prediction directly from H&E stained WSI. Our model was trained on WSI obtained from the TCGA dataset, where the EBV results were derived from RNA sequencing (RNA-seq). However, as EBER-ISH is a more common method to detect EBV status in practice^11,23^, a dataset labeled with EBER-ISH was used for fine-tuning to mitigate estimation bias and ensure robustness. To assess its generalizability, we externally validated our network on a dataset originating from a different institution.

## Results

### Patch-wise performance on TCGA dataset (internal validation)

**Figure 1:**
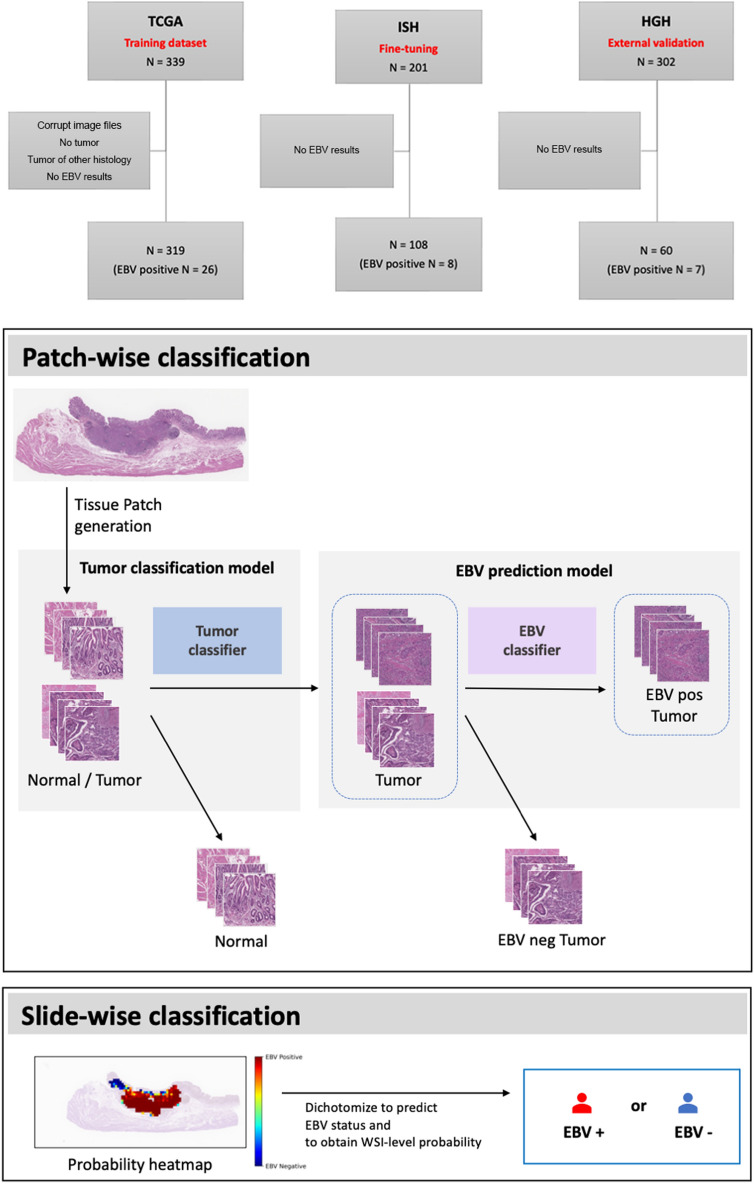
Overview of EBVNet. EBVNet was trained on the TCGA dataset, and fine-tuned on the ISH dataset. EBVNet before and after fine-tuning were externally validated on the HGH dataset. Each H&E histology slide was preprocessed by discarding non tissue-containing background using Otsu’s thresholding and generating non-overlapping patches. All the patches were stain normalized (not shown in the figure). First, the tumor classification model (blue box) receives all the tissue-containing patches and returns the probability of each patch being a tumor patch. The patches with probabilities of higher than 0.5 are assigned as tumor patches, otherwise, they are assigned as normal patches. The tumor-predicted patches are then fed into the second classifier (EBV prediction model, purple box), with the resulting probability of being an EBV positive tumor patch. The tumor-predicted patches with probabilities higher than 0.1 are assigned as EBV positive tumor patches, otherwise, they are assigned as EBV negative patches. Based on the patch-wise classification result, the EBV probability score (EPS), which is the ratio of the number of EBV-positive tumor-predicted patches to the number of tumor-predicted patches, is calculated for each slide. The slide with calculated EPS higher than 0.2 is assigned as an EBV positive slide, otherwise, it is assigned as an EBV negative slide.

The baseline framework has two sequential binary classifiers: a tumor classifier and an EBV classifier (Fig. 1). The performance of each classifier, trained using a different patch size and model, are shown in Table 1. To select the models for baseline framework, we compared the results of the internal validation using patch-wise performance on the hold-out TCGA dataset (Supplementary Table S3). As shown in Table 1, the models yielded better performance with a patch size of 512 × 512 pixels than with 256 × 256 pixels, while the computation time on larger images was longer than on smaller images (Supplementary Table S4).

**Table 1:** Patch-wise model performances in the tumor classifier (upper) and the EBV classifier (lower).

	Model	Patch size (pixels)	Accuracy	NPV^a^	Specificity	Sensitivity	Precision	F1-score
Tumor classifier	InceptionV3	256 × 256	0.98	0.97	0.96	0.98	0.98	0.98
		512 × 512	0.98	**0.99**	0.95	0.99	0.98	**0.99**
	ResNet50	256 × 256	0.98	0.97	0.96	0.99	0.98	0.98
		512 × 512	0.98	0.98	**0.97**	0.99	**0.99**	**0.99**
EBV classifier	InceptionV3	256 × 256	0.93	0.99	0.93	0.90	0.56	0.69
		512 × 512	**0.99**	0.99	**0.99**	**0.92**	**0.92**	**0.92**
	ResNet50	256 × 256	0.92	0.97	0.94	0.71	0.54	0.61
		512 × 512	0.98	0.99	**0.99**	0.91	0.86	0.89

Bold font: maximum value of specific metrics across different model and patch size.

^a^Negative predictive value.

We embedded the tumor classifier to select representative tumor patches. For the tumor classifier, we used ResNet50 because of its high specificity (Table 1). For the EBV classifier, we implemented InceptionV3, which yields higher sensitivity, since our aim was to enable our network to be used as a screening tool for EBV identification.

The confusion matrix, where the performance of the two classifiers was evaluated, is shown in Supplementary Figure S1. The baseline frameworks with different combinations of sequential binary classifiers were internally validated on the hold-out TCGA dataset, which was assessed with the 3-class classifier as well (Supplementary Fig. S2 and Supplementary Table S5). The results showed that when using the sequential binary classifiers, the false positive rate drastically decreased from 0.056 to 0.005 (Supplementary Figure S2 and Supplement table S5) and the overall performance was better in the sequential binary classifiers than in the 3-class classifier. The sequential binary classifiers with the best performance yielded a macro-negative predictive value (NPV) of 0.99 and a macro-sensitivity of 0.96.

### Representation visualization

**Figure 2:**
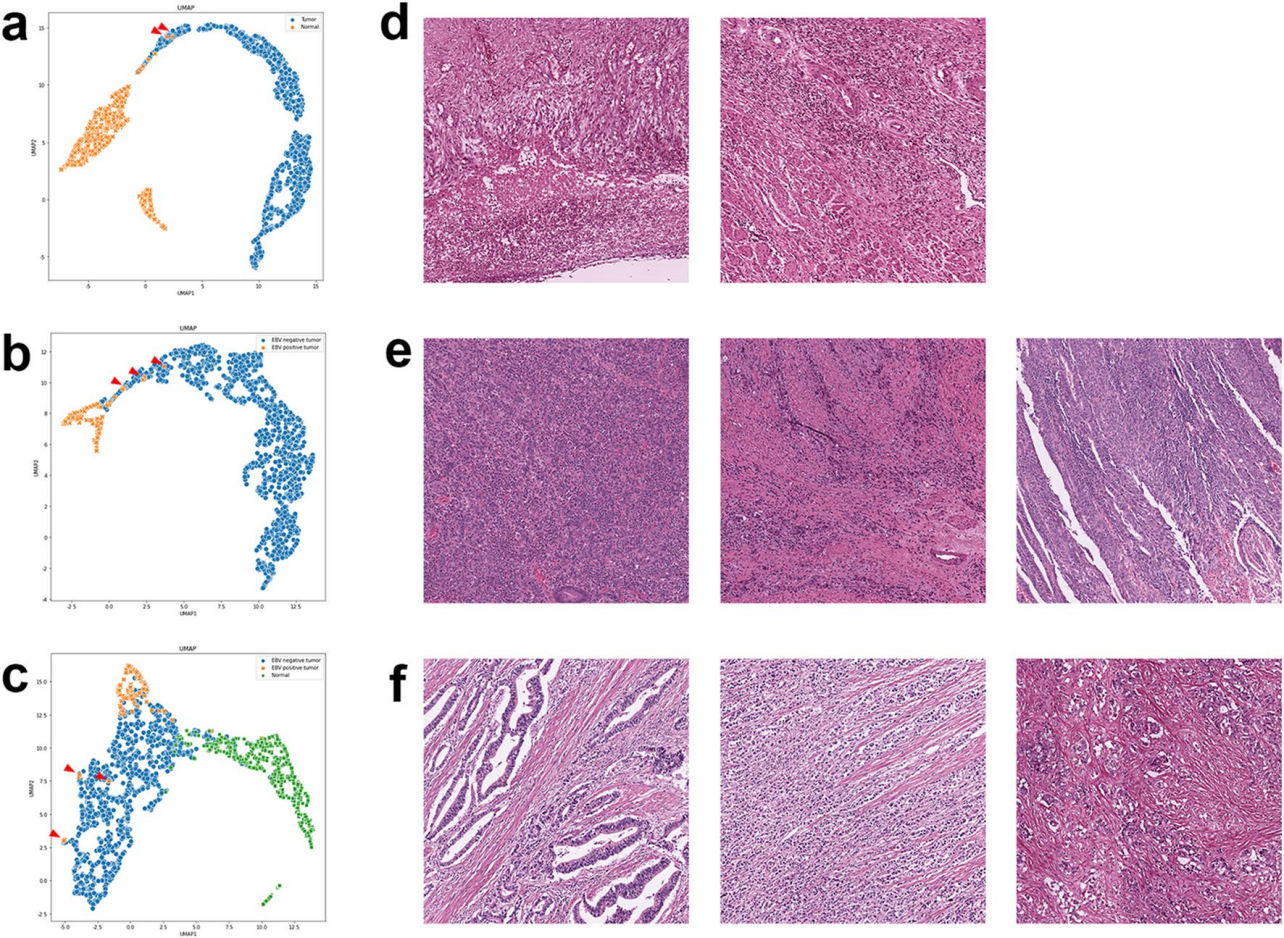
Uniform Manifold Approximation and Projection (UMAP) visualization of neural network latent space and examples of false results. All input patches were fed into classification models and the corresponding post-convolution dense vectors (1024-length) were generated. All the vectors were mapped into 2-dimensional space using UMAP. Each point corresponds to a patch in (**a**) the tumor classifier, (**b**) the EBV classifier, and (**c**) the 3-Class classifier, colored by tissue type (tumor vs. normal) and tumor type (EBV positive vs. EBV negative). Histology images **(d–f)** of false predictions (red triangles) of each classifier **(a–c),** which are located apart from their corresponding cluster on UMAP. These points were examined to investigate potential reasons for misclassification.

To have an insight into the model’s representation, we visualized the learned feature space in two dimensions using the hold-out TCGA dataset. The discriminative patches with similar features according to the classifiers were clustered closely in distinct regions, which proves the disentangled representation of each model (Fig. 2). Interestingly, several misclassified patches according to the binary classifiers are found at the edges of the other side cluster (Fig. 2a,b, arrows), whereas the misclassified patches predicted by the 3-class classifier are located far from their own cluster (Fig. 2c, arrows). Based on this result, it can be said that the binary classifiers learned the representation more efficiently than the 3-class classifier.

### Comparison of the baseline framework before and after fine-tuning (external validation based on slide-level performance)

**Figure 3:**
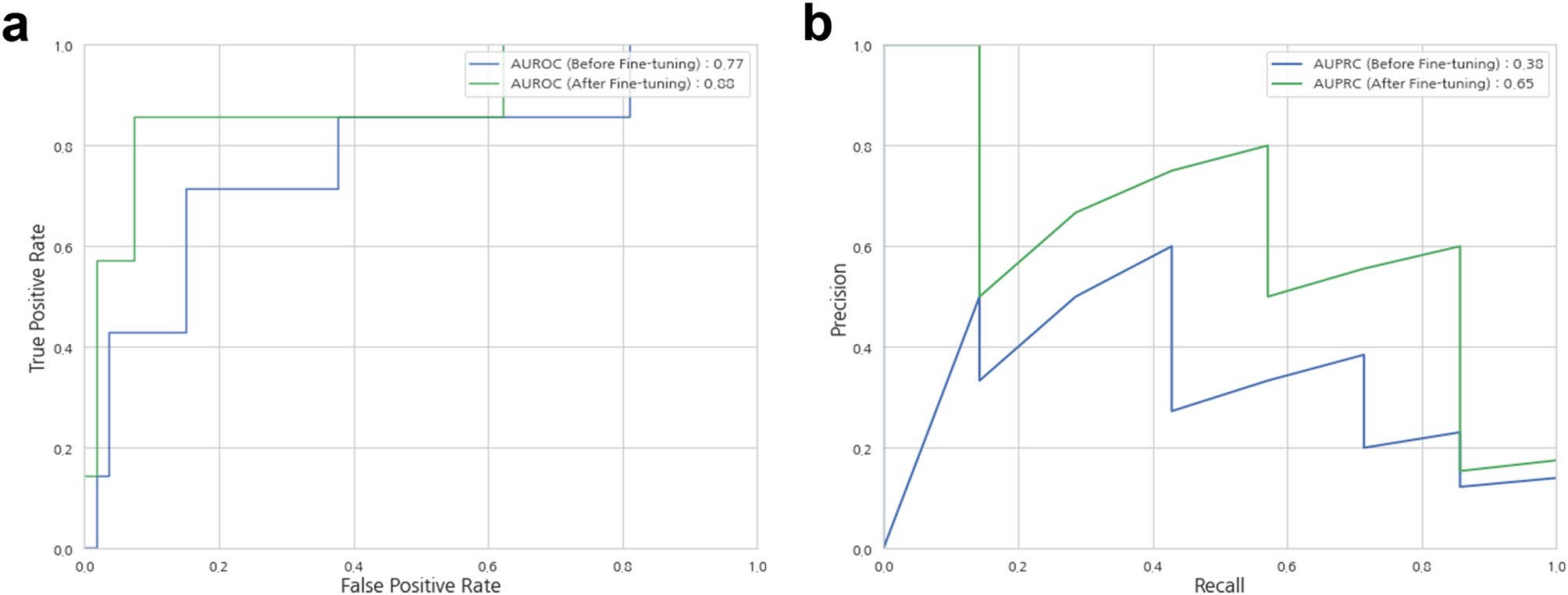
Performance of the models. The area under the receiver operating characteristic (AUROC) curve (**a**) and area under the precision-recall curve (AUPRC) (**b**) of slide-level EBV status inference using the original classifiers solely trained with TCGA dataset (blue) and re-weighted classifiers fine-tuned with ISH dataset (green).

In comparison with the slide-level performances using WSIs from Hanyang University Guri Hospital (Guri, South Korea; denoted by HGH), the performance after fine-tuning using WSIs from the International St. Mary’s Hospital (Incheon, South Korea; denoted by ISH) improved as follows: the accuracy increased from 0.77 to 0.92, the area under the receiver operating characteristic (AUROC) increased from 0.77 to 0.88, and the area under the precision-recall curve (AUPRC) increased from 0.38 to 0.65 (Table 2, Fig. 3).

**Table 2:** Slide-wise performance on the external dataset of the HGH cohort.

	Accuracy	NPV^a^	Specificity	Sensitivity	Precision	F1-score	AUROC^b^	AUPRC^c^
Before transfer learning	0.77	0.95	0.77	0.71	0.29	0.29	0.77	0.38
After transfer learning	0.92	0.98	0.92	0.86	0.60	0.71	0.88	0.65
Performance gain (value, %)	+ 0.15 (19.48)	+ 0.03 (3.16)	+ 0.15 (19.48)	+ 0.15 (21.13)	+ 0.31 (106.90)	+ 0.42 (144.83)	+ 0.11 (14.29)	+ 0.27 (71.05)

^a^Negative predictive value.

^b^The area under the receiver-operating characteristics curve.

^c^The area under the precision-recall curve.

**Figure 4:**
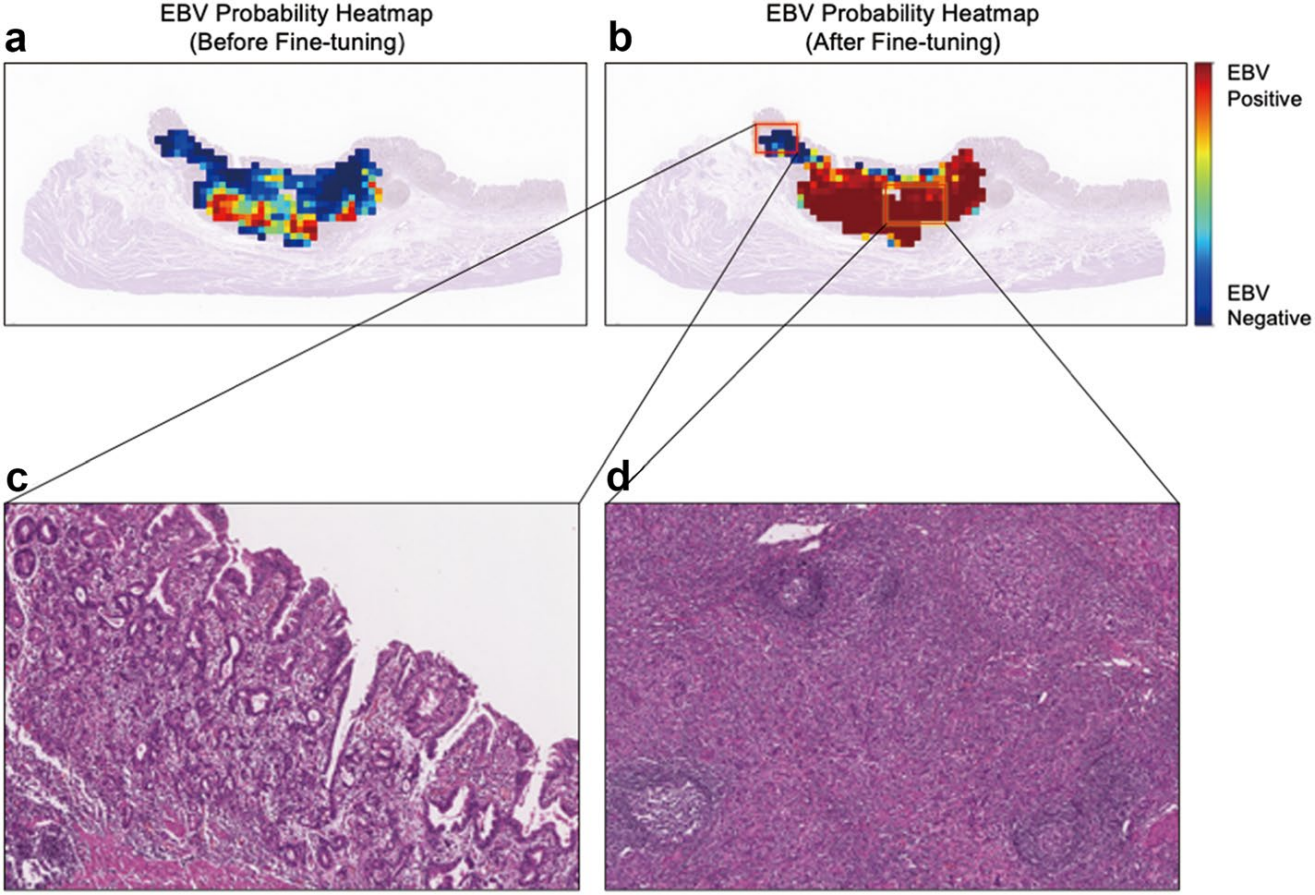
Representative heatmaps of an EBV positive case. The colors of the overlaid heatmaps represent the output of the EBV classification model (second classifier in the sequential classification framework), which means predicted probabilities of being an EBV-positive tumor, as defined in the color bar. In the top row, each heatmap corresponds to the EBV positive tumor probability values from classifiers before (**a**) and after (**b**) fine-tuning with the ISH dataset. A zoomed-in view of a region with low probability (**c**) and high probability (**d**) of being an EBV positive tumor, according to the fine-tuned EBV classifier (EBVNet), is visualized in the bottom row.

We implemented EBV probability heatmaps to visualize the effect of fine-tuning (Fig. 4a,b) and to identify the potential reasons for misclassification (Fig. 4c,d). After fine-tuning, regions with high probabilities of EBV-positive tumor tissue increased significantly (Fig. 4a,b). Tumors with a low probability for EBV (Fig. 4c) show differentiated histology, whereas the characteristic features of lymphoepithelial carcinoma were found with a high probability (Fig. 4d).

### Analysis of false results

We reviewed falsely predicted image patches on the hold-out TCGA test set (internal validation) (Fig. 2d–f). False-positive image patches by the tumor classifier include inflammatory necrotic tissue, florid granulation tissue, and dense infiltration of inflammatory cells, which could be mistaken as poorly-differentiated adenocarcinoma (Fig. 2d). False-negative tumor patches that were EBV positive but predicted as EBV negative by the EBV classifier and 3-class classifiers show a lack of lymphoepithelial features (Fig. 2e,f).

EBVNet identified one false negative and four false positives on the slide-wise HGH external validation set (Supplementary Figs. S3 and S4). The false negative exhibits differentiated histology with well-formed tubules (Supplementary Fig. S3). Of the four false positives, the algorithm falsely identified prominent lymphoid stroma and lymphoepithelial carcinoma (Supplementary Fig. S4A–C), and poorly cohesive carcinoma (Supplementary Fig. S4D), as EBV-positive tumors.

### Comparison of deep-learning model to pathologists based on slide-level performance

The performance results of all four pathologists were below the models’ AUROC and AUPRC, with a mean AUROC of 0.75 and a mean AUPRC of 0.41 (Supplementary Table S7). While the NPV and specificity were similar, EBVNet exhibited higher sensitivity than the pathologists.

## Discussion

In this study, we present EBVNet, a deep learning model to predict EBV status directly from histological images. Our model achieved higher performance than the network without fine-tuning, and outperformed experienced pathologists on a reader study. Our study also validated the feasibility of fine-tuning for domain adaptation and model generalizability.

Until now, seven studies on EBV status prediction via a deep learning approach using digitalized WSIs have been published (Supplementary Table S1)^25–31^. Of these, the most similar to our pipeline are those proposed by Zhang et al. and Zheng et al.^28,29^; in these two previous studies, a two-step approach utilizing a tumor classifier and an EBV classifier was implemented. Most studies have trained EBV classifiers using tumor patches generated from tumor annotation, with^28,29^ or without^25–27^ training tumor classifiers—a tumor annotation-based approach. Other studies have trained the model without using tumor or normal patches—an annotation-free training approach^30,31^. The latter is called weakly supervised learning where only a slide-level label (weak label) per image was available for model development^30,31^. Of the studies with weakly supervised learning, Muti et al. reported a comparable performance with an AUROC of 0.859 on the external dataset. However, the results of these previous studies elucidate that supervised learning approaches, where the model was trained on tumor patches, outperformed the weakly supervised approach with an AUROC of 0.941^29^.

Most histology-based deep learning studies implemented patch-based workflow due to hardware memory constraints. Patch selection techniques that take meaningful representative images for network training are paramount for higher network performance^55^. In the current study, we assumed that normal patches cannot provide any information about the genetic trait of the tumor. Therefore, we only used tumor patches based on the tumor annotation dataset when training the EBV classifier, similar to previous studies^25–29^. The tumor classifier in our proposed framework may have been useful in selecting representative patches (tumor patches) and in removing noise images of normal patches. We elucidated that the implementation of this patch selection network in sequential binary classifiers led to better performance than with a 3-class classifier.

However, training a discriminative model with supervision using tumor annotation has two drawbacks. First, annotation is a laborious, time-consuming, and expensive task, requiring domain knowledge and expertise. Second, detection of biomarkers using a model trained on annotated data is restricted to the detection of tumor regions that are already known. In contrast, unsupervised or weakly supervised learning allows the identification of novel biomarkers that are biologically relevant to the target of the model^56,57^. In the recent study by Brockmoeller et al., the model was trained on slide-level labels using tumor and non-tumor regions, and the image biomarker in the normal area, not in the tumor area, was first identified to predict lymph node metastasis^56^.

The increasing growth of deep learning owes plenty to “open data mentality”, with researchers sharing their datasets and code. Nevertheless, there is a lack of annotation data in histology-based deep learning, as the annotation of WSI is expensive and time-consuming. We would like to support further open-source development by sharing the annotations of TCGA-STAD employed in this study (https://github.com/EBVNET/EBVNET).

Our study has certain limitations that require further analysis. First, our proposed pipeline employed a fixed pooling operator for a slide-level aggregation. This simple decision fusion method of aggregating patch-level labels to a slide-level label is inconsistent with the decision process in pathology. In addition, this approach disregards spatial relationships between patches, resulting in a loss of global contextual information present in WSI. However, since EBV-positive tumors have morphologically homogeneous characteristics, simple thresholding or major voting, which are the most widely used approaches for the post-processing strategies of classification tasks, would have been effective^55^. Second, the use of EBV identification as a screening tool is more helpful on biopsy specimens, where lesions are much smaller and likely to be ignored. However, we validated EBVNet on the external dataset of HGH, which consists of WSI from gastrectomy specimens (Supplementary Table S6). The performance of our pipeline should be further fine-tuned and investigated on the hard cases of these biopsy slides. Finally, our network should be ameliorated for clinical applications achieving 100% sensitivity with an acceptable false rate. A large dataset for training or fine-tuning will be helpful to achieve this.

In conclusion, our study has illuminated the potential of deep learning systems to identify histology-based biomarkers. Our EBVNet is expected to serve as an alternative to effectively screen EBV status using ubiquitously available H&E slides.

## Methods

### Experimental design

In this study, three different datasets were used for training, fine-tuning, and external validation, respectively (Fig. 1). EBVNet, which consisted of the tumor classifier and EBV classifier (Fig. 1), was trained on a public dataset (TCGA-STAD). For fine-tuning and external validation, we used two datasets from different institutes, the ISH and HGH (Supplementary Table S6). The ISH dataset was employed for fine-tuning, and the HGH dataset for external validation. We also compared the performance of EBVNet before and after fine-tuning using slide-wise performance on the HGH dataset.

### Data acquisition and annotation

We retrieved anonymized histology images (diagnostic slides, FFPE tissue) from the TCGA-STAD project through the Genomic Data Commons Portal^58^. A total 319 WSI were used to train the deep learning model after removal of WSI with corrupt image files, lack of visible tumor tissue, missing values in EBER-RNA results, or histology for tumors other than adenocarcinoma (Fig. 1).

For fine-tuning, 108 WSI of gastric cancer were collected from ISH. To externally validate the developed model, we used an independent cohort that encompasses 60 WSI of stomach cancer with surgical resection from HGH.

All slides from the ISH and HGH cohorts were scanned at 40× objective magnification (∼0.25 μm/pixel) using Leica Aperio ScanScope AT2 (Leica Biosystems, Wetzlar, Germany). This study was approved by the Institutional Review Board at ISH (IRB no., IS21SIME0031) and HGH (IRB no., 2020-09-002), respectively, and conducted in accordance with the Declaration of Helsinki. Informed consent from patients was waived with IRB approval.

Digitalized H&E histology slides were re-surveyed, and adenocarcinoma regions in the TCGA and the ISH dataset were manually annotated by an expert gastrointestinal pathologist (S. Ahn) using the ASAP software, v.1.9.0 (Geert Litjens, Nijmegen, Netherlands). Every region with adenocarcinoma was assigned as “tumor.” As all WSIs used in this study contained tumor areas, which also have areas of normal tissue, the remaining unannotated areas were considered as normal tissue. We generated tumor patches based on tumor annotation, and normal patches derived from unannotated areas (Supplementary Table S3). Randomly selected patches generated from the TCGA datasets and not used in training classifiers, were used for internal validation (the hold-out TCGA dataset).

Full details for the strategies of image preprocessing, including patch generation, are provided in the Supplementary method.

### EBV labels

For TCGA samples, the EBV status was retrieved directly from the molecular result, as described by Liu et al. and Tathiane et al.^9,59^ without re-analysis for EBV. For samples in the ISH and HGH cohorts, EBV status was determined via EBER-ISH results.

### Model development

Deep neural networks, denoted by EBVNet, were trained on image patches with the aim of predicting EBV status in gastric cancers. The baseline framework consists of two sequential binary classifiers (Fig. 1). The first binary classifier is a tumor classification model (Normal vs. Tumor), and it is followed by the second binary classifier, which is the EBV prediction model (EBV negative tumor vs. EBV positive tumor). Among all the patches fed to the first classifier, only the patches predicted as tumors enter the second classifier. For training binary classifiers, two networks were used: ResNet50 and Inception V3. The ResNet network is well known for its residual connections and therefore used to mitigate the vanishing gradient and stabilize the learning process. The inception network consists of inception blocks with different parallel convolution filters for the extraction of representative features with fewer parameters. As an alternative approach to the sequential binary classifiers, we conducted an experiment to train a simple multi-classification network for EBV positive tumor, EBV negative tumor, and normal tissues, denoted by a 3-class classifier. For the 3-class classifier, Inception V3 was employed.

Limited generalizability of a deep learning model may hinder its clinical applicability resulting in poor performance on real-world data. One way to mitigate this problem is to re-weigh the model on target datasets with domain-specific features. Following this approach, fine-tuning can be used to ensure domain adaptation. Herein, the weight values of each binary classifier trained on the source TCGA dataset were employed to fine-tune the ISH dataset, as the ISH and HGH cohorts exhibited similar clinical characteristics and color spaces (Supplementary Table S6). All the layers were unfrozen and re-trained using the ISH dataset.

Full details on neural network training, model selection, hyperparameter optimization and data augmentation, are provided in the Supplementary method.

### Inference of EBV status

For patch-wise EBV status inference in a sequential binary classifier framework, first, patch images were given as input to the tumor classification model (Fig. 1), which then provided the probability of each patch being a tumor patch as output. The patches with predicted probabilities higher than 0.5 were assigned as tumor-predicted patches (N_tumor). Second, tumor-predicted patches were given as input to the EBV prediction model, which then provided the probability of each patch being an EBV positive tumor patch as output. The patches with predicted probabilities higher than 0.1 were assigned as EBV positive-predicted patches (N_EBVpos). Unlike the 0.5 threshold value of the tumor classifier, the cut-off for the EBV classifier was set to the low value of 0.1 to ensure that the sensitivity is high when detecting an EBV positive tumor patch. For inference in a 3-class classifier framework, patch images were given as input to the classification model (Normal vs. EBV negative tumor vs. EBV positive tumor), which then returned the probabilities of each class as output. The class with the highest probability was assigned as the prediction for each patch.

For slide-wise classification (EBV positive slide vs. EBV negative slide) after the patch-wise EBV status inference, we defined the EBV probability score (EPS) as the ratio of EBV positive-predicted patches to tumor-predicted patches (N_EBVpos / N_tumor). The cut-off value for EPS was calculated using Youden’s J statistic^60^, which is the cut-off value with the highest difference between true positive and false positive rate. The cut-off value was calculated for the HGH dataset as 0.2.

### Internal and external validation

The binary classifiers (the tumor classifier and the EBV classifier) and the 3-class classifier trained on the TCGA dataset were internally validated on the hold-out TCGA dataset using patch-wise performance.

We externally validated the performance of EBVNet on the HGH dataset. Slide-wise performance was assessed through the dichotomized outcomes of WSI in EBV status. The performance of EBVNet before fine-tuning was also compared to that after fine-tuning.

### Feature visualization

To visualize and interpret the deep learning predictions, we employed three approaches. First, we identified the falsely predicted patches for each class of the TCGA dataset, allowing observers to identify potential reasons for misclassification. Second, in order to appreciate the variability of learned representation, we used the three classifiers to generate and visualize post-convolution layer activation for the hold-out TCGA dataset. Activation values in the post-convolution layer were calculated on each image patch. Once calculated, activation vectors across all patches were mapped with the dimensionality reduction technique known as Uniform Manifold Approximation and Projection (UMAP)^61^. Finally, we rendered patch-level predictions for EBV status as activation maps, visualizing prediction scores as heatmap overlays on the original WSI in the HGH cohort.

### Reader study

To compare the performance of EBVNet with that of pathologists, we conducted a reader study in which four board-certified pathologists with 4–20 years of experience (JJ, JP, JS, and S Ahn) reviewed 168 WSI from both cohorts. The pathologists were blind to all clinical information, as well as EBVNet’s performance. For each WSI, they recorded whether they thought the cancer could be classified as EBV positive or EBV negative.

### Evaluation metrics

We compared the performance of each algorithm trained on the TCGA dataset, using patch-wise accuracy, sensitivity, specificity, NPV, precision, and F1-score. We measured the slide-wise performance of our network on HGH dataset using the AUPRC, AUROC, accuracy, sensitivity, specificity, NPV, precision, and F1-score. These metrics were defined as follows:

$$\mathrm{Accuracy }= \frac{TP+TN}{TP+FN+FP+TN},$$

$$\mathrm{Sensitivity }\left(\mathrm{Recall}\right)= \frac{TP}{TP+FN},$$

$$\mathrm{Specificity }= \frac{TN}{FP+TN},$$

$$\mathrm{Precision }= \frac{TP}{TP+FP},$$

$$\mathrm{Negative\, predictive \,values }\left(\mathrm{NPV}\right)= \frac{TN}{FN+TN},$$

$$\text{F1-score }= \frac{2\,\cdot\, precision\,\cdot \,recall}{precision\,+\,recall},$$

where TP, TN, FP, FN represented the total number of true positive, true negative, false positive, and false negative, respectively.

## Supplementary Information

Supplementary Information.

Supplementary Figures.

Supplementary Tables.

## Data Availability

The data that support the findings of this study were derived in part from TCGA database (https://www.cancer.gov/tcga) and their annotation are provided via an open-source license at https://github.com/EBVNET/EBVNET.

## Code availability

All source codes are available under an open-source license at https://github.com/EBVNET/EBVNET.
