# Supplementary material for: Deep learning model to predict Epstein–Barr virus associated gastric cancer in histology
Source: Sci Rep. 2022 Nov 2;12:18466. doi: 10.1038/s41598-022-22731-x (PMC9630260; doi:10.1038/s41598-022-22731-x)
Supplement: Supplementary file 1 — Supplementary Information. [file 41598_2022_22731_MOESM1_ESM.docx]

**Image preprocessing**

To remove non-tissue white background, a threshold-based segmentation method was adopted using the Otsu algorithm after the WSI were transferred to the HSV color space ^[1]^.

Next, the WSI were subdivided into non-overlapping image patches at a resolution of 1.0 μm/pixel, with a patch size of 1,024 x 1,024 pixels. Only patches with a tissue/background ratio of 0.7 were selected. Normal class patches were generated on the principle that even a minimal amount of tumor tissue is not acceptable. Patches where the tumor tissue accounts for more than 70% of area were classified as tumor class patches. These thresholds for patch generation were selected empirically using the TCGA dataset prior to fine-tuning and external validation, with the threshold adequacy verified by a pathologist (S. Ahn). The tumor class patches were allocated into “EBV positive” and “EBV negative” categories, based on the EBV label of each slide.

The generated patches were further curated to ensure that no tumor patches are mislabeled as normal, or vice versa. All patches were screened by the same pathologist (S. Ahn), and any erroneously classified patches were removed. Using this strategy of patch generation and curation, 40,126 patches were extracted from the TCGA datasets (Supplementary Table S2).

Using an algorithm developed by Vahadane ^[2]^, we conducted stain normalization with a reference image of the ISH dataset to normalize the TCGA dataset’s color style. The aforementioned strategy for patch generation was then applied to the ISH dataset, without curation and stain normalization. Approximately 10,000 patches were generated from the ISH dataset (Supplementary Table S2), and input into the fine-tuning stage.

**Neural network training, model selection, hyperparameter optimization and data augmentation**

During training neural networks, a base model (e.g., ResNet50, InceptionV3) was followed by a global average pooling layer and two fully-connected layers: one with 1,024 default units, and one with two or three dense units, depending on which classifiers are used. For binary classifiers in sequential models, the sigmoid function was used as the final activation function. Each prediction corresponds to a probability of being in a “positive” class (Tumor class in tumor classifier, EBV positive class in EBV classifier). In the case of the 3-class classifier, the softmax function was used as the final activation function. Each prediction means a probability of being in corresponding classes.

The dropout regularization was applied to the global average pooling layer and the first fully-connected layer (dropout ratio set to 0.5 as default). All training experiments were implemented within the Tensorflow framework ^[3]^.

The performance of the trained classifiers was evaluated on various convolutional neural networks (CNNs) and patch sizes (Table 1).

The weight values trained on the source TCGA dataset were employed to fine-tune the target ISH dataset, as the ISH and HGH cohorts exhibited similar clinical characteristics and color spaces (Supplementary Table S6). All layers were unfrozen and re-trained with the ISH dataset.

We trained the network using Adam optimization, a mini-batch size of 32, a dropout rate of 0.3, and global average pooling. The initial learning rates for the tumor and EBV classifiers were 1e−4 and 1e−3, respectively.

For all experiments, we compared the performance of the default model with that of the hyperparameter-tuned models. Hyperparameter tuning was conducted by using a Hyperband algorithm from the KerasTuner library (<https://github.com/keras-team/keras-tuner>) ^[4,5]^ to select the optimal set of hyperparameters. The sampled hyperparameter space was as follows: learning rates of 1e−2, 1e−3, 1e−4, and 1e−5: Adam and SGD optimizers; post-convolution pooling of “max” and “average”; dropout rates of 0.3, 0.4, 0.5, and 0.6; unit sizes of the first fully connected layer following the base model from 32 to 2,048 with increments of 32. Each set of hyperparameters was trained with maximum 10 epoch, with the number of models trained in every iteration reduced by one-third. The hyperparameter set that exhibited minimum validation loss was then selected as the optimal set. Between the default and hyperparameter-tuned architecture, we used the architecture with better test performance.

To avoid overfitting, data was augmented using the Albumentation open-source library ([https://github.com/albumentations- team/albumentations/](https://github.com/albumentations-%20team/albumentations/)) ^[6]^. The following transformations were applied: horizontal and vertical flip, elastic transformation, color shift of hue, and saturation and brightness value. Each operation was applied to the training images with a probability of 0.5.

**References**

1. Otsu, N. A threshold selection method from gray-level histograms. *IEEE Transactions on Systems, Man, and Cybernetics* **9**, 62-66 (1979).

2. Vahadane, A. *et al.* Structure-preserving color normalization and sparse stain separation for histological images. *IEEE Trans Med Imaging* **35**, 1962-1971 (2016).

3. Abadi, M. *et al.* TensorFlow: a system for large-scale machine learning. Type of Work at <https://arxiv.org/abs/1605.08695> (2016).

4. Li, L. S., Jamieson, K., DeSalvo, G., Rostamizadeh, A. & Talwalkar, A. Hyperband: a novel bandit-based approach to hyperparameter optimization. [publication in English]. *J Mach Learn Res* **18**, 1-52 (2018).

5. Manaswi, N. K. Understanding and working with keras in *Deep Learning with Applications Using Python* (ed. Manaswi, N. K.) Ch. Chapter 2, 31-43 (Apress, 2018).

6. Buslaev, A. *et al.* Albumentations: fast and flexible image augmentations. *Information* **11**, 125 (2020).
