# Supplementary material for: Deep learning model to predict Epstein–Barr virus associated gastric cancer in histology
Source: Sci Rep. 2022 Nov 2;12:18466. doi: 10.1038/s41598-022-22731-x (PMC9630260; doi:10.1038/s41598-022-22731-x)
Supplement: Supplementary file 2 — Supplementary Figures. [file 41598_2022_22731_MOESM2_ESM.docx]

**Supplementary Figure S1**

**
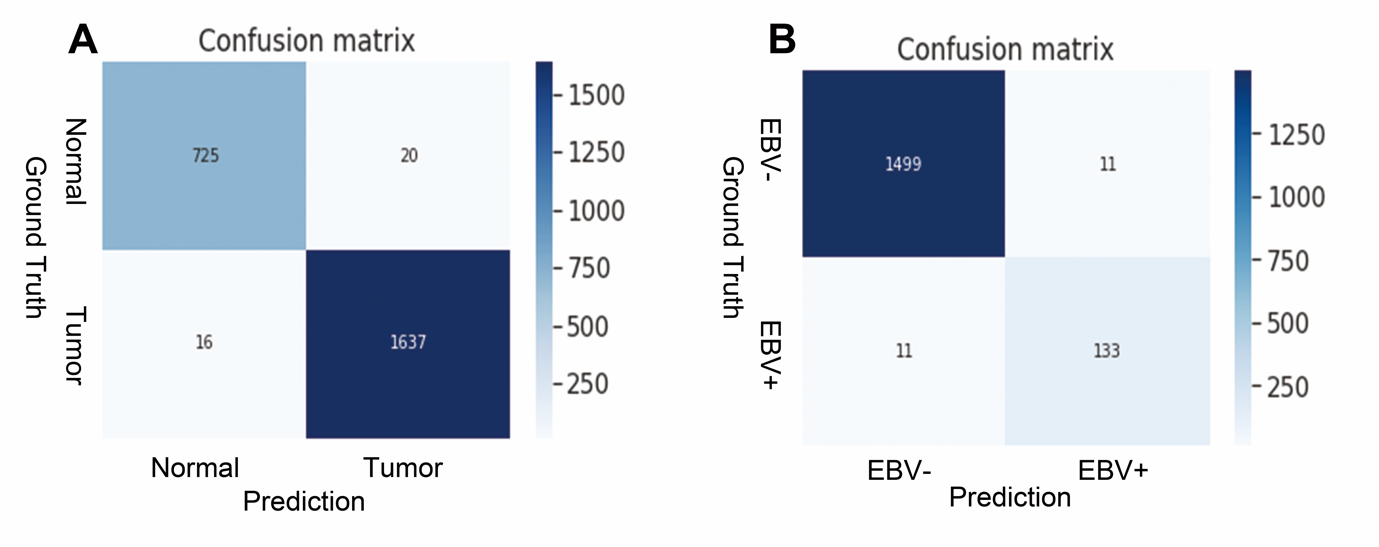
**

**Supplementary Figure S1.** Confusion matrix (A) for ground-truth tumor and prediction by the tumor classifier, and (B) for ground-truth EBV status and prediction by the EBV classifier on the hold-out TCGA set.

**Supplementary Figure S2**


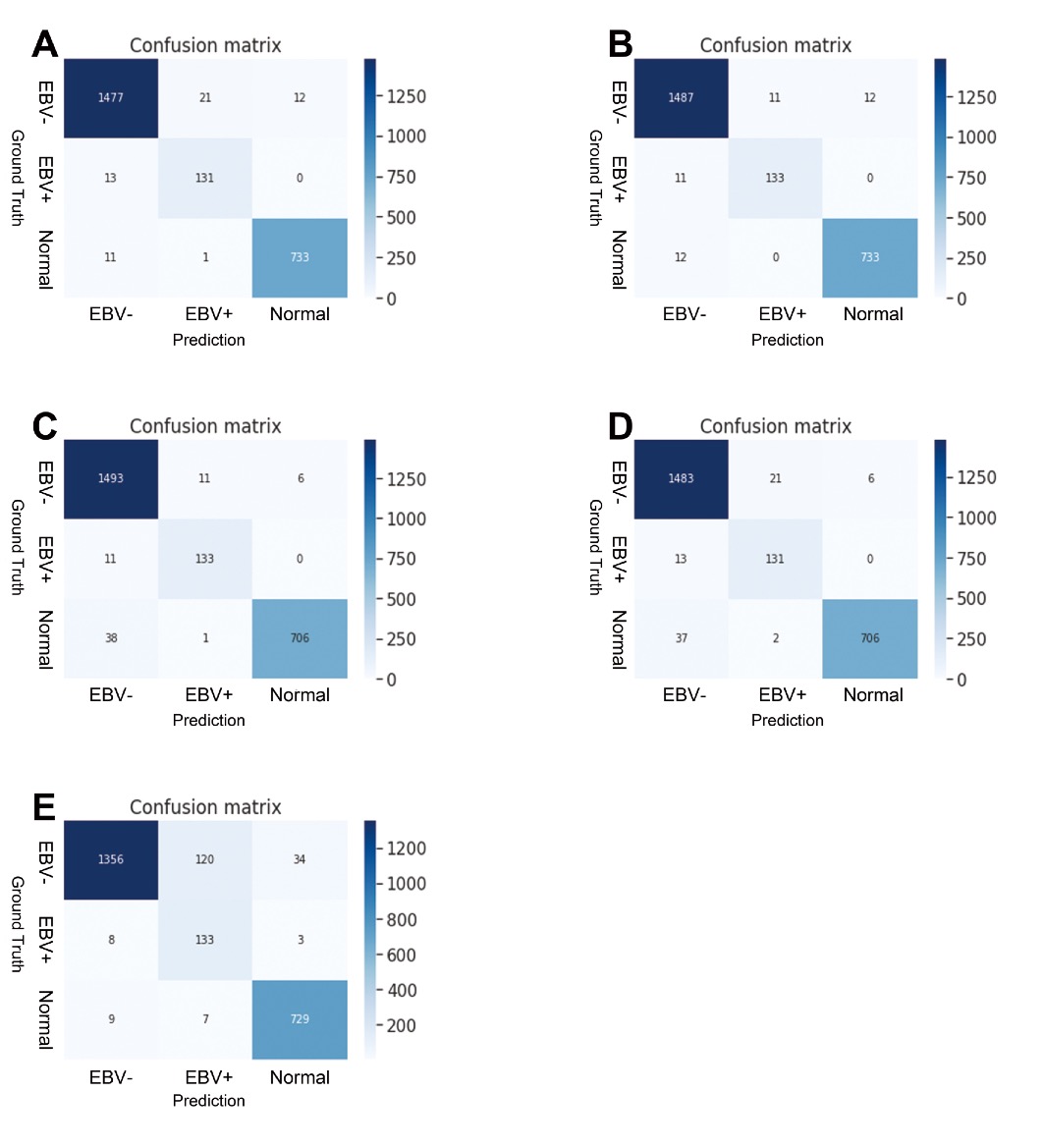


**Supplementary Figure S2.** Confusion matrix for ground-truth tumor and prediction (A-D) by the sequential binary classifiers and (E) by the 3-class classifier on the hold-out TCGA set. The combinations of tumor and EBV classifiers are as follows: (A) ResNet50 / ResNet50; (B) ResNet50 / InceptionV3; (C) InceptionV3 / InceptionV3; (D) InceptionV3 / ResNet50. For the sequential binary classifiers (A-D), the threshold value for the tumor classification model (Normal vs. Tumor) was 0.5, and the threshold value for the EBV prediction model (EBV negative tumor vs. EBV positive tumor) was 0.1. For the 3-class classifier (E), patches were assigned to the class with the highest probability from the softmax function.

**Supplementary Figure S3**


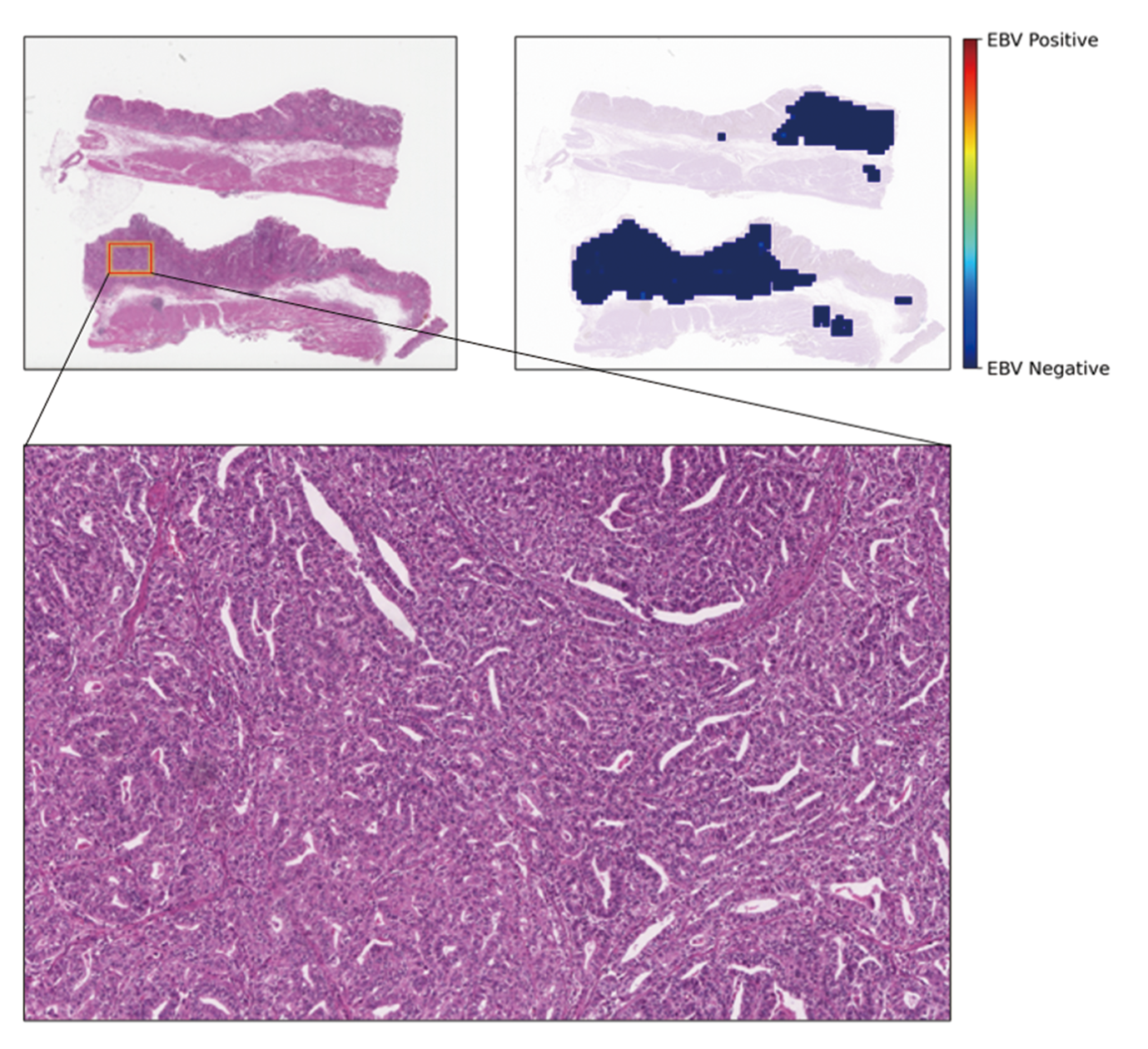


**Supplementary Figure S3.** A case of false-negative prediction of external validation with potential reasons for misclassification.

**Supplementary Figure S4A**


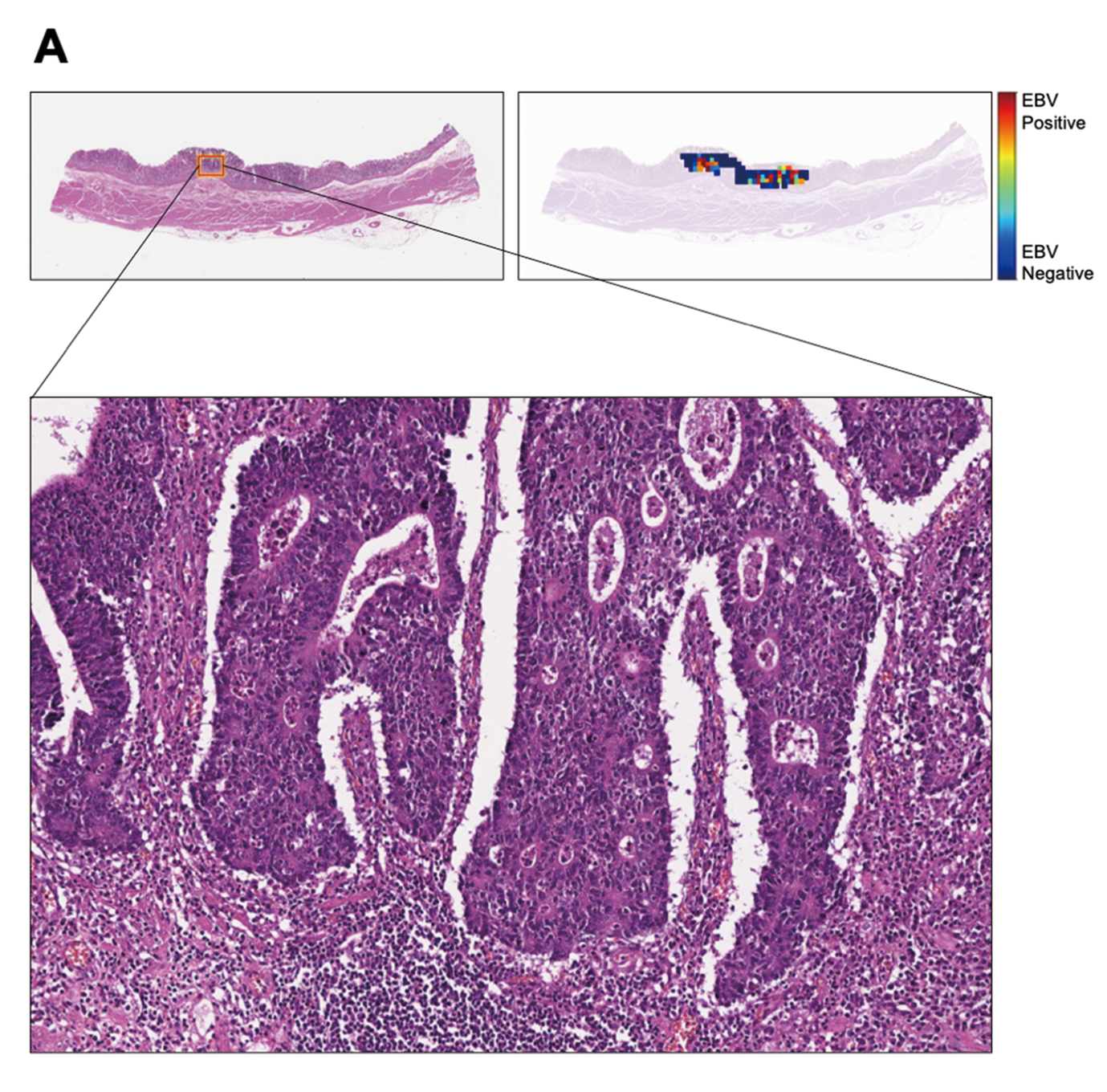


**Supplementary Figure S4B**


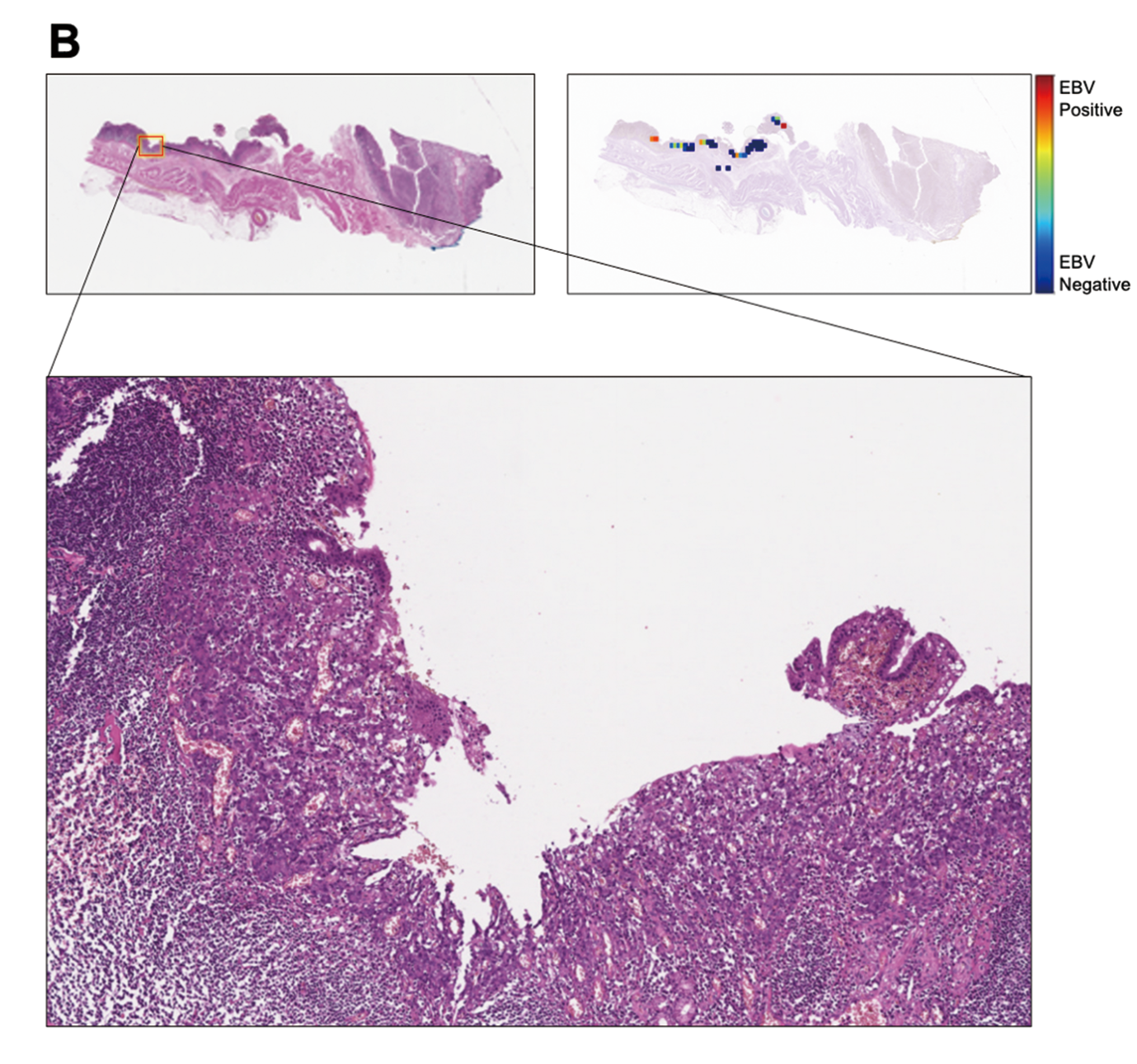


**Supplementary Figure S4C**


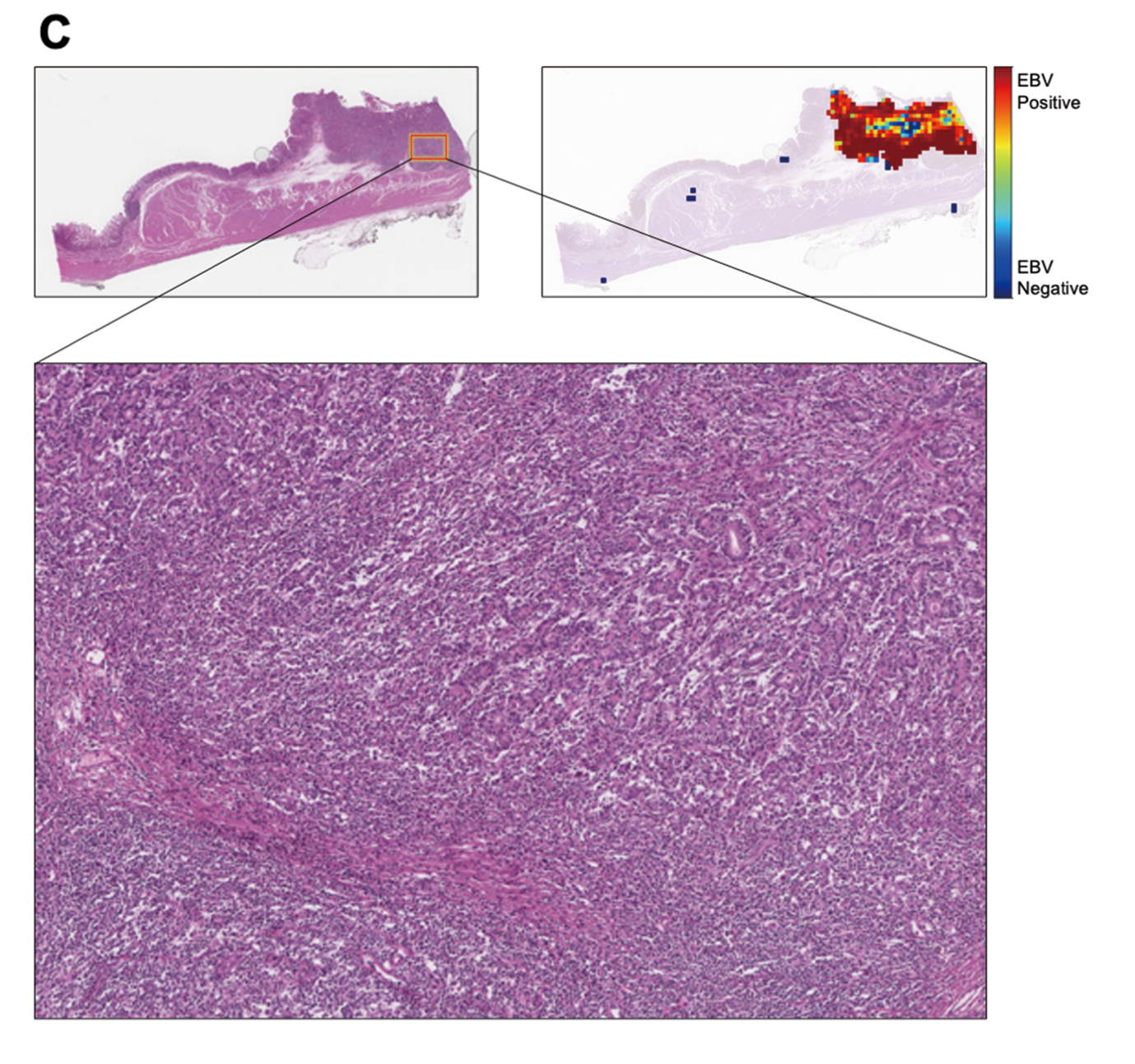


**Supplementary Figure S4D**


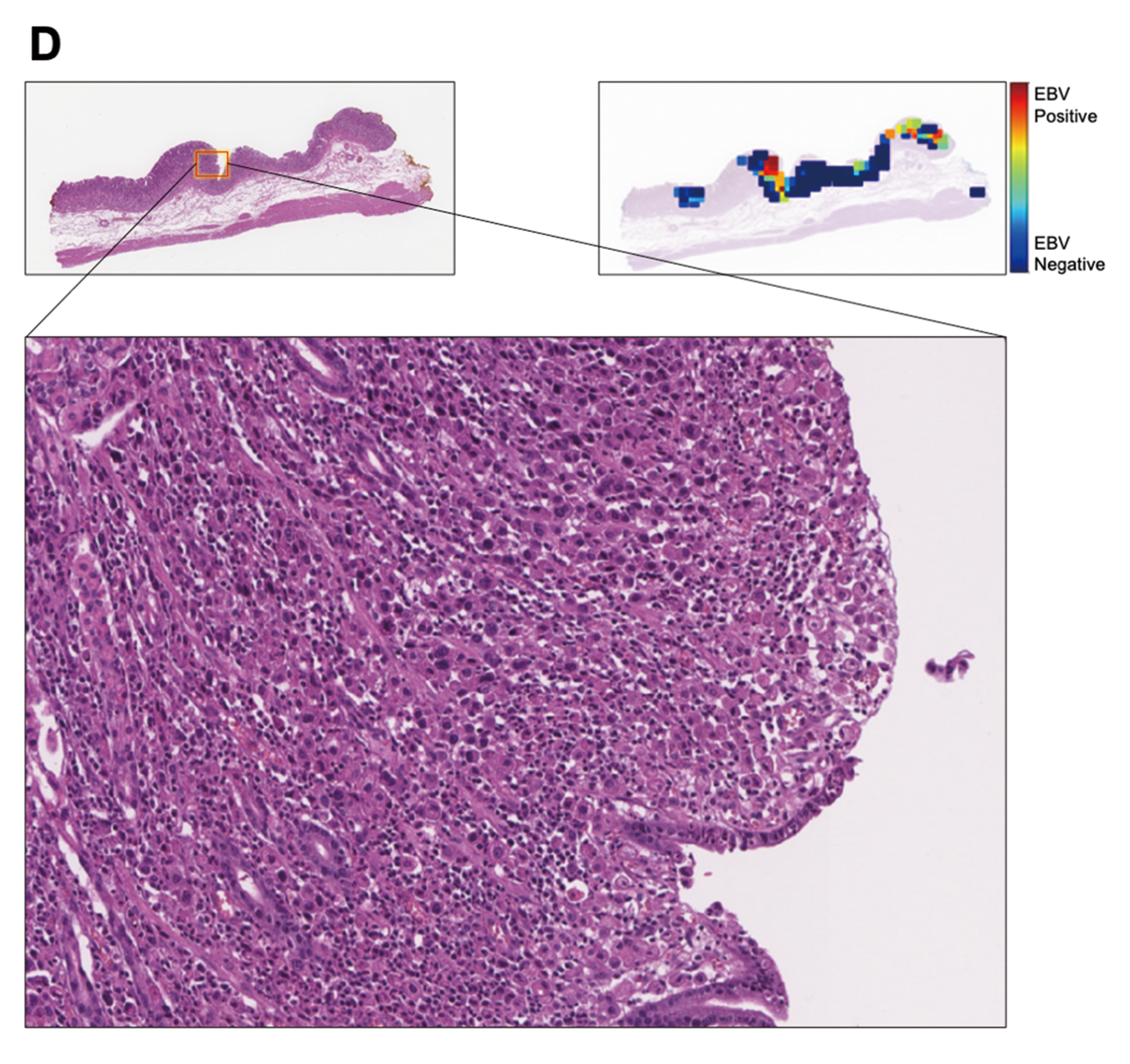


**Supplementary Figure S4.** Four cases of false-positive prediction of external validation with potential reasons for misclassification.
