# Supplementary material for: Deep learning model to predict Epstein–Barr virus associated gastric cancer in histology
Source: Sci Rep. 2022 Nov 2;12:18466. doi: 10.1038/s41598-022-22731-x (PMC9630260; doi:10.1038/s41598-022-22731-x)
Supplement: Supplementary file 3 — Supplementary Tables. [file 41598_2022_22731_MOESM3_ESM.docx]

Supplementary Table S1. Previous histology-based deep learning approaches to predict EBV status in gastric cancer^1-7^

| No.  / Study  (year) | Neural Network  Architecture | Patch size  (Pixels) | Collected data | Target | Neural Network Training / Evaluation Strategy | Performance |
| --- | --- | --- | --- | --- | --- | --- |
| 1  / Flinner et al.,  (2022) | DenseNet161 | 600 x 600 | **for model development**  TCGA-STAD dataset  (total 133 pts)  - 27 EBV  - 38 MSI  - 32 GS  - 36 CIN  **for external validation**  UKC images  (total 538 pts)  - 25 EBV  - 44 MSI  - 42 GS  - 427 CIN | - 4-class  : EBV vs. MSI vs. GS vs. CIN | Select 100 image patches from cancerous regions for each WSI  Train individual CNNs using a bagging-like approach (randomly assign patients to datasets)  Make ensembled prediction by simple consensus / majority vote over all individual CNNs | Patient level  AUC 0.68 |
| 2  / Hinata et al.,  (2021) | VGG16  VGG19  ResNet50  EfficientNet B0 | 224 x 224 | **for model development**  UTokyo cohort  (total 408 pts)  - 108 EBV  - 58 MSI  - 242 others  **for external validation**  TCGA-STAD  (total 244 pts)  - 23 EBV+  - 44 MSI  - 177 others | - EBV + MSI vs. others  - EBV vs. MSI vs. others  - EBV vs. MSI + others  - MSI vs. EBV + others | Randomly select 256 images from each case and calculate output probability  Then aggregate 256 results via average and adopt the class corresponding to the highest value | AUC 0.756 - 0.870  EBV + MSI vs. others |
| 3  / Kather et al, (2019) | VGG19 | 224 x 224 | **for model development**  TCGA-STAD  (total 317 pts)  - 26 EBV Positive  **for external validation**  KCCH-STAD cohort  (total 197 pts)  - 10 EBV Positive | - 2-class  : EBV positive vs. EBV negative |  | AUC 0.81 |
| 4  / Zhang et al.,  (2021) | ResNet18 | 512 x 512 | TCGA-STAD dataset  (total 122 pts)  - 24 EBV positive | - 3-class  : tumor vs. dense normal vs. loose normal  - 2-class  : EBV positive vs. EBV negative | Tumor vs. normal model (TN model) was first applied to distinguish tumor and normal tiles  EBV+ vs. EBV-model (EBV model) was trained on tumor and normal tiles separately to predict EBV status  Sample level EBV score is calculated by proportion of predicted EBV+ tiles across all tumor or normal tiles from each sample | Patch level  AUC 0.85 |
| 5  / Zheng et al.,  (2022) | ResNet50 | 512 x 512 | **for model development**  Internal-STAD  (total 1,006 WSIs)  - 203 EBV positive  **for external validation**  MultiCenter-STAD  (total 417 WSIs)  - 98 EBV positive  TCGA-STAD dataset  (total 234 WSIs)  - 24 EBV positive | - 2-class  : EBV positive vs. EBV negative | Tumor detector was first trained  EBVNet (EBV + vs EBV -) was trained on tumor patches | AUC  Internal-STAD 0.969  External-STAD 0.941  TCGA-STAD 0.895 |
| 6  / Muti et al.,  (2021) | ShuffleNet | 512 x 512 | 10 cohorts  (total 2,685 pts) | - 2-class  : EBV positive vs. EBV negative | Train model within each cohort  Train model within pooled training dataset (from 5 largest cohort), then validated on each remaining cohorts  Three-way classification  (EBV positive vs. microsatellite instable vs. double-negative tumours)  Classification performance  : stratified by tumor-to-total tissue ratio of each slide  low (0-0.33), medium (0.34-0.66), high (>0.66) | AUC  KCCH 0.836  AUGSB 0.672  ITALIAN 0.859  TUM 0.676 |
| 7  / Laleh et al.,  (2022) | ResNet  EfficientNet  Vision Transformers  (ViT)  Mutiple instance learning (MIL)  Attention-based MIL  (AttMIL)  Clustering constrained attention MIL (CLAM) | 512 x 512 | **for model development**  BERN cohort  (total 304 pts)  - 8 EBV Positive  **for external validation**  TCGA-STAD cohort  (total 327 pts)  - 26 EBV Positive | - 2-class  : EBV positive vs.  EBV negative | MIL : Uses maximum pooling mechanism for single high scoring tile to predict the label of bag  AttMIL : Uses attention score of each tile to calculate the bag level feature vector, then predicts the label of bag using this vector  CLAM : Uses attention-based pooling for aggregating tile-level features into slide-level representations | AUC  ResNet 0.779  EfficientNet 0.787  ViT 0.775  MIL 0.795  AttlMIL 0.732  CLAM 0.813 |

Supplementary table S2. Previous histology-based deep learning studies to infer the genetic traits^8-27^

| No. | Study (year) | Methods | Class of Target molecules | Tumor type | Collected data | Performance |
| --- | --- | --- | --- | --- | --- | --- |
| 1 | Kather et al.,  (2020) | VGG19 | Virus (HPV, EBV) | Head and neck cancer (HPV) Gastric cancer (EBV) | HPV classifier  - train: 412 pts^a^ (pos: 12 %) - test: 105 pts (pos: 49 %) EBV classifier - train: 412 pts (pos: 8 %) - test: 197 pts (pos: 5 %) | AUC for HPV classifier : 0.70 [0.66, 0.74] AUC for EBV classifier : 0.81 [0.69, 0.89] |
| 2 | Kather et al.,  (2019) | ResNet18 | Microsatellite instability | Gastric and colon cancer | Three domain datasets - TCGA: 315 pts (STAD), 738 pts (CRC) - DACHS: 378 pts - KCCH: 185 pos | AUC 0.60 - 0.92 |
| 3 | Xu et al.,  (2018) | Xception | Tumor mutation burden | Bladder cancer | 253 pts | AUC 0.75 |
| 4 | Couture et al.,  (2018) | VGG16 | Molecular subtype | Breast cancer | train: 571 pts test: 288 pts | Accuracy  - intrinsic subtype: 77 - ROR-PT status: 76 - Histologic subtype: 94 |
| 5 | Schaumberg et al.,  (2018) | ResNet50 | *SPOP* mutation | Prostate cancer | Two domain datasets - TCGA: 499 pts (pos: 35.5 %) - MSK-IMPACT: 138 pts (pos: 13.8%) | AUC 0.86 |
| 6 | Coudry et al.,  (2018) | InceptionV3 | Gene mutation (*STK11, EGFR, FAT1, SETBP1, KRAS, TP53*) | Lung cancer | 1,635 WSIs | AUC 0.73 - 0.86 |
| 7 | Liu et al.,  (2020) | GAN based augmentation,  ResNet50 | *IDH* mutation | Brain tumor | 266 pts | AUC 0.931 |
| 8 | Schmauch et al.,  (2020) | Multilayer perceptron | Gene expression | 28 solid tumor types | 8,725 pts | AUC 0.63 - 0.82 |
| 9 | Kather et al.,  (2020) | ResNet-18 AlexNet Inception-V3 DenseNet-201 ShuffleNet | Single gene mutation  Molecular subtype | 14 solid tumor types | Two domain datasets - TCGA  - DACHS: 408 pts | AUC - Single gene mutation: 0.60 - 0.78 - *BRAF:* 0.74 - 0.78 |
| 10 | Sirinukunwattana et al.,  (2020) | InceptionV3 +  Domain adversarial neural network | Molecular subtype | Colon cancer | Three domain datasets - FOCUS: 666 WSIs - TCGA: 463 WSIs  - GRAMPIAN: 406 WSIs | AUC, macro-average - FOCUS: 0.90 [0.87, 0.91] - TCGA: 0.84 [0.8, 0.87] - GRAMPIAN : 0.85 [0.82, 0.89] |
| 11 | Jang et al.,  (2020) | InceptionV3 | Gene mutation (*APC, KRAS, PIK3CA, SMAD4, TP53)* | Colon cancer | Two domain datasets - TCGA: 629 pts - GRAMPIAN: 142 pts | AUC 0.693 - 0.809 |
| 12 | Kim et al.,  (2019) | InceptionV3 | *BRAF, NRAS* mutation | Melanoma | 324 WSIs (266 pts) | AUC - *NRAS*: 0.92 [0.81, 1.00] - *BRAF*: 0.83 [0.45, 1.00] |
| 13 | Sun et al.,  (2019) | DenseNet-121 | *BAP1* mutation | Uveal melanoma | 47 pts | AUC 0.99 |
| 14 | Sha et al.,  (2019) | Multi FOV DL : ResNet18 | *PD-L1* | Lung cancer | 130 WSIs | AUC 0.67 - 0.81 |
| 15 | Yamashita et al.,  (2021) | MSINet : ResNet34  (tumor classifier) + MobileNetV2  (MSI classifier) | Microsatellite instability | Colon cancer | train: 100 WSIs test: 484 WSIs | AUC 0.931 [0.771, 1.000] |
| 16 | Xu et al.,  (2021) | DenseNet-121 | Chromosomal instability | Breast cancer | 1,065 WSIs (1,010 pts) | AUC 0.822 |
| 17 | Fu et al.,  (2020) | Modified InceptionV4 | All types of genomic alteration | 28 solid tumor types | 17,355 WSIs (10,452 pts) | AUC 0.63 - 0.82 |
| 18 | Zhang et al.,  (2019) | Modified CNN | Tumor mutation burden | Liver cancer | 368 WSIs | patch-wise AUC 0.9488 |
| 19 | Chen et al.,  (2021) | Slide-level assessment model (SLAM) | *BRAF, MSI* | Colon cancer | Two domain datasets - DACHS: 3,279 pts - YCR-BCIP: 889 pts | AUC  - *BRAF*: 0.821 [0.786, 0.852] - MSI: 0.909 [0.899, 0.929] |
| 20 | Bilal et al.,  (2021) | Iterative draw-and-rank sampling  (IDaRS) | Molecular subtype | Colon cancer | Two domain datasets - TCGA: 497 pts - PAIP: 47 pts | AUC 0.73 - 0.86 |

^a^ patients: [ ] Confidence interval

Supplementary table S3. Composition patch dataset in the TCGA and ISH cohort

| Class | | TCGA | | | ISH | |
| --- | --- | --- | --- | --- | --- | --- |
|  |  | **Training** | **Validation** | **Test** ^a^ | **Training** | **Validation** |
| Tumor | **EBV positive** | 8,120 | 2,328 | 144 | 952 | 408 |
|  | **EBV negative** | 10,670 | 3,063 | 1,510 | 2,102 | 901 |
| Normal | | 10,524 | 3,022 | 745 | 4,040 | 1,733 |
| Total | | 29,314 | 8,413 | 2,399 | 7,094 | 3,042 |

^a^ the hold-out TCGA dataset

Supplementary table S4. Comparison of computing time for each model training

|  | No. of patches | Network | Patch size (Pixels) | Batch Size | Computing Time  (Seconds per 1 epoch) |
| --- | --- | --- | --- | --- | --- |
| Tumor  classifier | 22,209 | InceptionV3 | 256 x 256 | 128 | 1,614 |
|  |  |  | 512 x 512 | 32 | 4,166 |
|  |  | ResNet50 | 256 x 256 | 128 | 1,636 |
|  |  |  | 512 x 512 | 32 | 4,127 |
| EBV  classifier | 18,790 | InceptionV3 | 256 x 256 | 128 | 1,406 |
|  |  |  | 512 x 512 | 32 | 3,462 |
|  |  | ResNet50 | 256 x 256 | 128 | 1,385 |
|  |  |  | 512 x 512 | 32 | 3,517 |

Supplementary table S5. Patch-wise performance with the application of different combinations to baseline framework

|  | | Sequential Binary classifiers  (Tumor classifier / EBV classifier) | | | | 3-Class classifier |
| --- | --- | --- | --- | --- | --- | --- |
|  |  | ResNet50  /ResNet50 | ResNet50  /InceptionV3 | InceptionV3  /InceptionV3 | InceptionV3  / ResNet50 |  |
| EBV Positive | **Accuracy** | 0.985 | 0.991 | 0.990 | 0.985 | 0.942 |
|  | **Negative Predictive value** | 0.994 | 0.995 | 0.995 | 0.994 | 0.995 |
|  | **Sensitivity** | 0.910 | 0.924 | 0.924 | 0.910 | 0.924 |
|  | **Specificity** | 0.990 | 0.995 | 0.995 | 0.990 | 0.944 |
|  | **Precision** | 0.856 | 0.924 | 0.917 | 0.851 | 0.512 |
|  | **F1 Score** | 0.882 | 0.924 | 0.920 | 0.879 | 0.658 |
|  | **False Positive Rate** | 0.010 | 0.005 | 0.005 | 0.010 | 0.056 |
|  | **False Negative Rate** | 0.090 | 0.076 | 0.076 | 0.090 | 0.076 |
| EBV Negative | **Accuracy** | 0.976 | 0.981 | 0.972 | 0.968 | 0.929 |
|  | **Negative Predictive value** | 0.963 | 0.963 | 0.980 | 0.969 | 0.852 |
|  | **Sensitivity** | 0.978 | 0.985 | 0.989 | 0.982 | 0.898 |
|  | **Specificity** | 0.973 | 0.974 | 0.945 | 0.944 | 0.981 |
|  | **Precision** | 0.984 | 0.985 | 0.968 | 0.967 | 0.988 |
|  | **F1 Score** | 0.981 | 0.985 | 0.978 | 0.975 | 0.941 |
|  | **False Positive Rate** | 0.027 | 0.026 | 0.055 | 0.056 | 0.019 |
|  | **False Negative Rate** | 0.022 | 0.015 | 0.011 | 0.018 | 0.102 |

Supplementary table S6. Patients and tumor characteristics for the image analysis data set

|  | TCGA  Training set (n = 319) N (% ^a^) | ISH  Training set  (n = 108) N (%^a^) | HGH  Validation set (n = 60) N (%^a^) | Chi-square *P* value |
| --- | --- | --- | --- | --- |
| Age |  |  |  | 0.557 |
| < 50 years | 25 (7.8) | 6 (5.6) | 3 (5.0) |  |
| > 50 years | 288 (90.3) | 102 (94.4) | 57 (95.0) |  |
| missing | 6 (1.9) | 0 | 0 |  |
| Sex |  |  |  | 0.470 |
| Male | 195 (61.1) | 82 (75.9) | 17 (28.3) |  |
| Female | 85 (26.6) | 26 (24.1) | 43 (71.7) |  |
| Missing | 39 (12.2) | 0 | 0 |  |
| Tumor location |  |  |  | <0.001 |
| Upper third | 62 (19.4) | 13 (12.0) | 1 (1.7) |  |
| Middle third | 95 (29.8) | 18 (16.7) | 11 (18.3) |  |
| Lower third | 116 (36.4) | 73 (67.6) | 48 (80.0) |  |
| Indetermined | 0 | 4 (3.7) | 0 |  |
| Missing | 7 (2.2) | 0 | 0 |  |
| Tumor gross |  |  |  | 0.044 ^d^ |
| Elevated | NA ^b^ | 31 (28.7) | 7 (11.7) |  |
| Flat | NA | 20 (18.5) | 12 (20.0) |  |
| Depressed | NA | 57 (52.8) | 40 (66.7) |  |
| Missing |  |  | 1 (1.7) |  |
| Tumor size (mm) |  |  |  | 0.070 ^d^ |
| Median (range) | NA | 36.73 (5-150) | 30.02 (3-95) |  |
| Missing | NA | 0 | 1 |  |
| Differentiation ^c^ |  |  |  | 0.005 |
| Differentiated | 157 (49.2) | 72 (66.7) | 29 (48.3) |  |
| Undifferentiated | 162 (50.8) | 36 (33.3) | 31 (51.7) |  |
| Lauren classification |  |  |  | 0.001 |
| Intestinal | 155 (48.6) | 72 (66.7) | 29 (48.3) |  |
| Diffuse | 90 (28.2) | 29 (26.9) | 14 (23.3) |  |
| Indeterminate | 74 (23.2) | 7 (6.5) | 17 (28.3) |  |
| Depth of invasion (pT) |  |  |  | <0.001 |
| pT1 | 12 (3.8) | 75 (69.4) | 60 (100.0) |  |
| pT2 | 67 (21.0) | 10 (9.3) | 0 |  |
| pT3 | 154 (48.3) | 7 (6.5) | 0 |  |
| pT4 | 84 (26.3) | 15 (13.9) | 0 |  |
| Missing | 2 (0.6) | 1 (0.9) | 0 |  |
| Lymph node status |  |  |  | <0.001 |
| Negative | 99 (31.0) | 55 (50.9) | 50 (83.3) |  |
| Positive | 217 (68.0) | 26 (24.1) | 10 (16.7) |  |
| Missing | 3 (0.9) | 27 (25.0) | 0 |  |
| EBV status |  |  |  | 0.608 |
| Negative | 293 (91.8) | 100 (92.6) | 53 (88.3) |  |
| Positive | 26 (8.2) | 8 (7.4) | 7 (11.7) |  |
|  |  |  |  |  |
| Procedure |  |  |  | <0.001 |
| Biopsy | 0 | 2 (1.9) | 0 |  |
| Resection | 319 (100) | 106 (98.1) | 60 (100.0) |  |

^a^ All percentage weighted for sampling design

^b^ Japanese classification of gastric cancer

^c^ Not available

^d^ t-test between ISH and HGH

Supplementary table S7. Pathologist sensitivity, specificity, negative predictive value, AUROC, and AUPRC on the reader study

|  | NPV ^a^ | Specificity | Sensitivity | F1-score | AUROC ^b^ | AUPRC ^c^ |
| --- | --- | --- | --- | --- | --- | --- |
| Pathologist 1 | 0.964  [0.929, 0.983] | 0.862  [0.798, 0.911] | 0.706  [0.440, 0.897] | 0.471  [0.321, 0.607] | 0.659  [0.593-0.729] | 0.374  [0.260-0.507] |
| Pathologist 2 | 0.968  [0.936, 0.985] | 0.962  [0.920, 0.986] | 0.706  [0.440, 0.897] | 0.686  [0.516, 0.821] | 0.818  [0.720-0.904] | 0.505  [0.311-0.703] |
| Pathologist 3 | 0.961  [0.929, 0.979] | 0.943  [0.895, 0.974] | 0.647  [0.383, 0.858] | 0.595  [0.414, 0.739] | 0.756  [0.662-0.847] | 0.407  [0.237-0.590] |
| Pathologist 4 | 0.950  [0.920, 0.969] | 0.956  [0.911, 0.982] | 0.530  [0.278, 0.770] | 0.546  [0.348, 0.703] | 0.756  [0.652-0.862] | 0.338  [0.174-0.533] |

^a^ Negative predictive value; ^b^ the area under the receiver-operating characteristics curve; ^c^ the area under the precision-recall curve

[ ] Confidence interval

**References**

1. Flinner, N.*, et al.* Deep learning based on hematoxylin-eosin staining outperforms immunohistochemistry in predicting molecular subtypes of gastric adenocarcinoma. *J Pathol* **257**, 218-226 (2022).

2. Hinata, M. & Ushiku, T. Detecting immunotherapy-sensitive subtype in gastric cancer using histologic image-based deep learning. *Sci Rep* **11**, 22636 (2021).

3. Kather, J.N.*, et al.* Deep learning detects virus presence in cancer histology. *BioRxiv*, 690206 (2019).

4. Zhang, B., Yao, K., Xu, M., Wu, J. & Cheng, C. Deep Learning Predicts EBV Status in Gastric Cancer Based on Spatial Patterns of Lymphocyte Infiltration. *Cancers (Basel)* **13**(2021).

5. Zheng, X.*, et al.* A deep learning model and human-machine fusion for prediction of EBV-associated gastric cancer from histopathology. *Nat Commun* **13**, 2790 (2022).

6. Muti, H.S.*, et al.* Development and validation of deep learning classifiers to detect Epstein-Barr virus and microsatellite instability status in gastric cancer: a retrospective multicentre cohort study. *Lancet Digit Health* **3**, e654-e664 (2021).

7. Ghaffari Laleh, N.*, et al.* Benchmarking weakly-supervised deep learning pipelines for whole slide classification in computational pathology. *Med Image Anal* **79**, 102474 (2022).

8. Coudray, N.*, et al.* Classification and mutation prediction from non-small cell lung cancer histopathology images using deep learning. *Nat. Med.* **24**, 1559-1567 (2018).

9. Kather, J.N.*, et al.* Deep learning can predict microsatellite instability directly from histology in gastrointestinal cancer. *Nat. Med.* **25**, 1054-1056 (2019).

10. Liu, S.*, et al.* Isocitrate dehydrogenase (IDH) status prediction in histopathology images of gliomas using deep learning. *Sci. Rep.* **10**, 7733 (2020).

11. Schmauch, B.*, et al.* A deep learning model to predict RNA-Seq expression of tumours from whole slide images. *Nat. Commun.* **11**, 3877 (2020).

12. Kather, J.N.*, et al.* Pan-cancer image-based detection of clinically actionable genetic alterations. *Nat Cancer* **1**, 789-799 (2020).

13. Sirinukunwattana, K.*, et al.* Image-based consensus molecular subtype (imCMS) classification of colorectal cancer using deep learning. *Gut* **70**, 544-554 (2021).

14. Jang, H.J., Lee, A., Kang, J., Song, I.H. & Lee, S.H. Prediction of clinically actionable genetic alterations from colorectal cancer histopathology images using deep learning. *World J. Gastroenterol.* **26**, 6207-6223 (2020).

15. Sun, M.*, et al.* Prediction of BAP1 expression in uveal melanoma using densely-connected deep classification networks. *Cancers (Basel)* **11**, 1579 (2019).

16. Sha, L.*, et al.* Multi-field-of-view deep learning model predicts non small cell lung cancer programmed death-ligand 1 status from whole-slide hematoxylin and eosin images. *J. Pathol. Inform.* **10**, 24 (2019).

17. Yamashita, R.*, et al.* Deep learning model for the prediction of microsatellite instability in colorectal cancer: a diagnostic study. *Lancet Oncol.* **22**, 132-141 (2021).

18. Xu, Z.*, et al.* Deep learning predicts chromosomal instability from histopathology images. *iScience* **24**, 102394 (2021).

19. Couture, H.D.*, et al.* Image analysis with deep learning to predict breast cancer grade, ER status, histologic subtype, and intrinsic subtype. *NPJ Breast Cancer* **4**, 30 (2018).

20. Schrammen, P.L.*, et al.* Weakly supervised annotation-free cancer detection and prediction of genotype in routine histopathology. *J. Pathol.* **256**, 50-60 (2022).

21. Schaumberg, A.J., Rubin, M.A. & Fuchs, T.J. H&E-stained whole slide image deep learning predicts SPOP mutation state in prostate cancer. (bioRxiv, 2018).

22. Kather, J.N.*, et al.* Deep learning detects virus presence in cancer histology. (bioRxiv, 2019).

23. Xu, H., Park, S., Lee, S.H. & Hwang, T. Using transfer learning on whole slide images to predict tumor mutational burden in bladder cancer patients. (bioRxiv, 2019).

24. Fu, Y.*, et al.* Pan-cancer computational histopathology reveals mutations, tumor composition and prognosis. *Nat Cancer* **1**, 800-810 (2020).

25. Kim, R.H.*, et al.* A deep learning approach for rapid mutational screening in melanoma. (bioRxiv, 2019).

26. Zhang, H.*, et al.* Predicting tumor mutational burden from liver cancer pathological images using convolutional neural network. in *2019 IEEE International Conference on Bioinformatics and Biomedicine (BIBM)* (eds. Yoo, I., Bi, J. & Hu, X.) 920-925 (IEEE, San Diego, CA, USA, 2019).

27. Bilal, M.*, et al.* Novel deep learning algorithm predicts the status of molecular pathways and key mutations in colorectal cancer from routine histology images. (medRxiv, 2021).
